# Economic Evaluation of Universal Lynch Syndrome Screening Protocols among Newly Diagnosed Patients with Colorectal Cancer

**DOI:** 10.3390/jpm11121284

**Published:** 2021-12-02

**Authors:** Jing Hao, Dina Hassen, James M. Gudgeon, Susan R. Snyder, Heather Hampel, Marc S. Williams, Ravi N. Sharaf, Christine Y. Lu, Janet L. Williams, Victoria Schlieder, Alanna Kulchak Rahm

**Affiliations:** 1 Department of Population Health Sciences, Geisinger, Danville, PA 17822, USA; dahassen@geisinger.edu; 2 Genomic Medicine Institute, Geisinger, Danville, PA 17822, USA; mswilliams1@geisinger.edu (M.S.W.); janetlw810@gmail.com (J.L.W.); akrahm@geisinger.edu (A.K.R.); 3 Precision Genomics, Intermountain Healthcare, Murray, UT 84107, USA; jimgudgeon@comcast.net; 4 Health Policy & Behavioral Sciences, School of Public Health, Georgia State University, Atlanta, GA 30303, USA; ssnyder2@gsu.edu; 5 Internal Medicine, The Ohio State University Wexner Medical Center, Columbus, OH 43210, USA; hhampel@coh.org; 6 Division of Gastroenterology, Department of Medicine and Healthcare Policy and Research, Weill Cornell Medicine, New York, NY 10021, USA; ras9030@med.cornell.edu; 7 Department of Population Medicine, Harvard Pilgrim Health Care Institute, Harvard Medical School, Boston, MA 02215, USA; christine_lu@harvardpilgrim.org; 8 Investigator Initiated Research Operations, Geisinger, Danville, PA 17822, USA; vmschlieder@geisinger.edu

**Keywords:** decision analysis, economic evaluation, Lynch syndrome screening, colorectal cancer

## Abstract

We conducted an updated economic evaluation, from a healthcare system perspective, to compare the relative effectiveness and efficiency of eight Lynch syndrome (LS) screening protocols among newly diagnosed colorectal cancer (CRC) patients. We developed decision analytic models for a hypothetical cohort of 1000 patients. Model assumptions and parameter values were based on literature and expert opinion. All costs were in 2018 USD. For identifying LS cases, the direct germline sequencing (DGS) protocol provided the best performance (sensitivity 99.90%, 99.57–99.93%; specificity 99.50%, 97.28–99.85%), followed by the tumor sequencing to germline sequencing (TSGS) protocol (sensitivity, 99.42%, 96.55–99.63%; specificity, 96.58%, 96.46–96.60%). The immunohistochemistry (IHC) protocol was most efficient at $20,082 per LS case identified, compared to microsatellite instability (MSI) ($22,988), DGS ($31,365), and TSGS ($104,394) protocols. Adding double-somatic testing to IHC and MSI protocols did not change sensitivity and specificity, increased costs by 6% and 3.5%, respectively, but reduced unexplained cases by 70% and 50%, respectively. DGS would be as efficient as the IHC protocol when the cost of germline sequencing declines under $368 indicating DGS could be an efficient option in the near future. Until then, IHC and MSI protocols with double-somatic testing would be the optimal choices.

## 1. Introduction

Precision medicine aims to improve individual health outcomes by tailoring healthcare based on genomic and all other relevant information [1]. One example is universal colorectal cancer (CRC) tumor screening for Lynch syndrome (LS) [2,3]. In the United States, CRC is the second leading cause of cancer deaths and about 148,000 new cases of CRC are expected to be diagnosed in 2020 [4]. LS is the most common form of inherited CRC accounting for 2 to 4 percent of these cancers [3]. Universal tumor screening for LS is an evidence-based, cost-effective strategy recommended by many professional organizations and national guidelines for all patients with CRC at diagnosis [2,5,6,7,8,9,10,11,12,13]. Identifying LS variants in patients with CRC allows them to benefit from intensive cancer screening and surveillance, treatment options (e.g., immunotherapy [14]), and the option for more extensive colonic surgery to decrease the risk of metachronous malignancy [15].

There are multiple LS screening strategies/protocols for LS patients with newly diagnosed CRC. Currently, LS cases are identified through immunohistochemical (IHC) staining or microsatellite instability (MSI) analysis conducted on pathology slides testing for presence (IHC) or the function (MSI) of the mismatch repair (MMR) proteins (*MLH1*, MLH2, MLH6, and PMS2) [3,16,17]. If the tumor has absent IHC staining for an MMR protein and/or is MSI-high, the tumor is considered to have defective mismatch repair (dMMR). If the *MLH1* protein is absent or if the tumor is MSI-high, further testing for methylation of the *MLH1* promoter and/or the somatic *BRAF* p.V600E variant are recommended. If either of these tests have positive results, LS is unlikely since these changes are almost always somatic. Patients with dMMR tumors without *MLH1* promoter methylation or *BRAF* variant are candidates for germline testing to establish a diagnosis of LS, as confirmed by presence of a pathogenic/likely pathogenic variant in one of the MMR genes.

Existing economic evaluation studies have focused on comparing IHC, MSI, and direct germline sequencing protocols [9,10,11,12,13]. Universal LS screening of all newly diagnosed CRC patients, or in those diagnosed under 70 years old, has been found to be cost-effective by most measures compared to no screening, or only screening of a sub-population (e.g., those with family history or younger than 50 years old) [12,13]. The studies consistently demonstrate that the IHC protocol is the most cost-effective and efficient protocol. Adding *MLH1* methylation and *BRAF* further improves the cost-effectiveness and efficiency [11,13]. Economic analysis of direct germline sequencing of all CRC patients never resulted in an acceptable cost-effectiveness threshold [9]. However, there are concerns with the traditional IHC and MSI protocols. False negative results are a limitation: IHC sensitivity is 83% for *MLH1*, *MSH2*, or *MSH6* variants, and MSI sensitivity is 87% for *MLH1* or *MSH2* variants and 77% for *MSH6* variants [3,18]. In addition, the implementation of LS screening in healthcare practice is suboptimal [19]. One of the many factors causing this is its complexity, involving multiple steps which can confuse patients and clinicians and cause potential loss of follow up of patients [18,20,21].

The need for revisiting the economic utility of universal LS screening protocols has risen in recent years. First, the price for germline genetic sequencing has declined dramatically. Second, new evidence of “double somatic” variants in the MMR genes resulting in dMMR in the tumor explains approximately 68% of nonmethylated MMR cases without a germline MMR variant [18,22,23,24] which accounts for 33% to 75% of patients with a dMMR CRC with unexplained MMR deficiency. In the past, these patients were typically treated as if they had LS without a detectable germline variant [17,25]. Third, availability and evidence of new testing including tumor next-generation sequencing (tumor sequencing) or tumor and germline paired analysis of MMR genes, which is simpler and showed superior sensitivity to current multi-test approaches [18], brings the possibility of a new LS screening approach. The new evidence may lead to replacement of currently recommended LS screening protocols with direct germline sequencing or upfront tumor sequencing of all newly diagnosed CRC patients.

The objective of this study was to conduct an updated economic analysis, from a healthcare system perspective, to evaluate and compare the relative effectiveness and efficiency of multiple LS screening protocols among newly diagnosed CRC patients that are deemed viable based on current evidence.

## 2. Materials and Methods

This study is part of a larger study, the IMPULSS (implementing universal Lynch syndrome screening) project [26].

### 2.1. Study Population

The study population to support modeling and simulation was a hypothetical cohort of 1000 patients with newly diagnosed CRC.

### 2.2. Model Development and Structure

**Figure 1:**
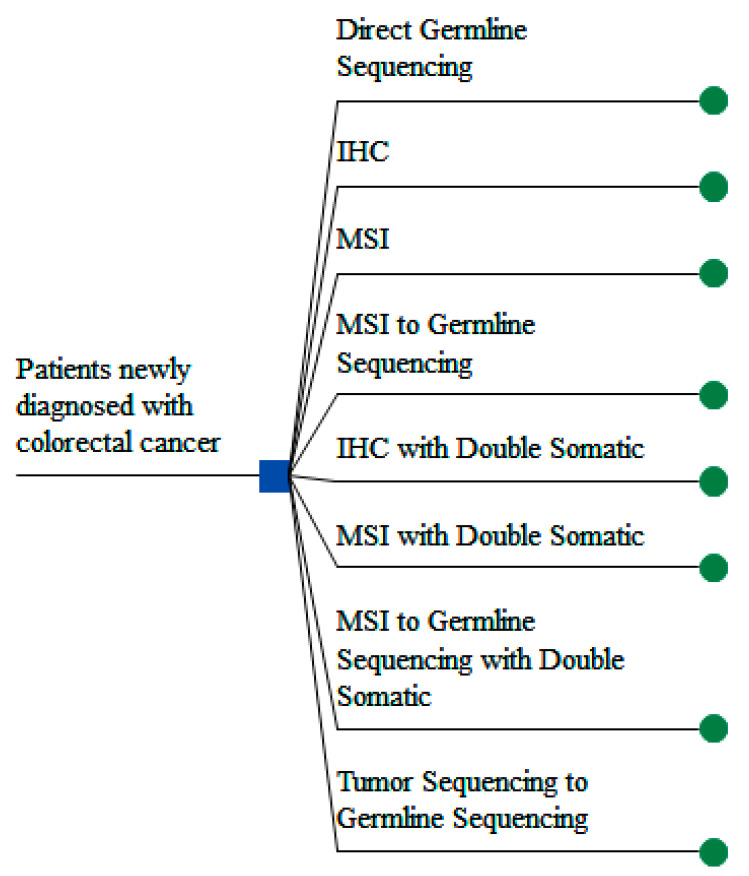
Lynch syndrome (LS) screening protocols. Note: IHC, immunohistochemistry; MSI, microsatellite instability. Germline sequencing refers to next generation sequencing (NGS) panel including MMR genes in all protocols.

We developed decision analytic models using decision trees to represent eight LS screening protocols for identifying LS cases among newly diagnosed CRC populations and to evaluate their relative effectiveness, costs, and efficiency from a healthcare system perspective (Figure 1). The eight modeled protocols are deemed viable to reflect current evidence and interests of healthcare systems based on input and consensus from the IMPULSS clinical expert panel which is teamed with the clinical experts of the IMPULSS study team from eight participating healthcare systems and the IMPULSS External Advisory Board (Supplementary Materials Figure S1).

### 2.3. Parameter Estimates

Table A1 (Appendix A) represents the full parameter table that presents base-case values, ranges, probability distributions, and references for all parameters. The parameters values were estimated based on literature, public sources including Medicare Fee Schedule, and expert opinion from the IMPULSS clinical expert panel. The prevalence of LS in CRC patients was based on national prevalence [3]. The probabilities of test results at each step of the protocols were modeled and simulated. All test sensitivity and specificity values in the models were based on the detection of LS cases. Since sensitivity and specificity values for *BRAF* and *MLH1* promoter hypermethylation tests reported in the literature refer to test performance in identifying somatic changes [3] and not in terms of detection of LS cases, we back-calculated the sensitivity and specificity values based on their positive predictive value (PPV) and negative predicted value (NPV) and prevalence of LS at relevant points in the models (Appendix B for more details).

We applied fair market prices to represent the costs from a healthcare system perspective. Most costs were obtained from the Medicare 2018 Fee Schedule and a range between 0.5–1.5 times the Medicare reimbursement amount was applied and adjudicated by expert opinion for sensitivity analysis. For cost of germline sequencing, we applied the patient price amount from two testing companies as the lower and higher bounds to reflect the wide range in cost of germline genetic sequencing in the current market [27,28]. All costs were reported in 2018 US dollars.

### 2.4. Model Assumptions

We assumed 100% availability and success in blood and tumor tissue collection, tests being successful and reportable, and 100% compliance with protocols (Appendix A Table A1). We also assumed genetic testing used in all protocols for detecting LS cases is a next-generation sequencing (NGS) panel including MMR genes, based on expert opinion that this is the testing most commonly used in practice and prices are similar for panel tests.

### 2.5. Outcome Measures

We compared effectiveness, costs, and efficiency across the eight modeled LS screening protocols. The main clinical outcome is the effectiveness where we calculated the protocol sensitivity (number of true positives for LS expected to be identified by the protocol/(prevalence*cohort size)) and specificity (number of true negatives for LS expected to be identified by the protocol/(cohort size-(prevalence*cohort size)). Based on the protocol sensitivity and specificity, we reported the number of true positive LS cases expected to be identified by the protocol and the number of expected missed (false negatives) LS cases. We also reported the number of unexplained dMMR cases. For costs, we reported total protocol costs for a hypothetical cohort of 1000 and costs per CRC case screened. Efficiency was calculated as the cost per true LS case identified.

### 2.6. Analyses Performed

A base-case analysis was performed using the best estimates (base-case values) for all model parameters and inputs. One-way sensitivity analyses were performed to assess the effects of changes in individual parameters on the estimated model outcomes demonstrated using tornado diagrams. Probabilistic sensitivity analyses were further performed with 10,000 iterations based on assigned probability distributions for each parameter to evaluate the plausible ranges (reported as 95% CI) for model outcomes. In addition, we conducted a threshold analysis to estimate the threshold cost of a germline genetic sequencing panel at which the direct germline sequencing (DGS) protocol would reach equivalent efficiency as the IHC protocol. We also estimated the threshold cost of tumor sequencing for the tumor sequencing to germline sequencing (TSGS) protocol to reach equivalent efficiency as the IHC protocol. The models were developed using Microsoft Excel, with the @RISK (Palisade Corporation, Newfield, NY, USA) add-on for conducting sensitivity and threshold analyses.

### 2.7. Validation

We conducted internal validation and checks for each of the models. External validations were performed by comparing our model outputs at various model points to values reported in the literature or values based on analysis from unpublished data. A full list of external validations conducted is included in Supplementary Materials Table S1.

## 3. Results

### 3.1. Base-Case and Sensitivity Analyses

The results from external validation are shown in Supplementary Materials Table S1. Table 1 summarizes outcome results from base–case and probabilistic sensitivity analysis. For protocol effectiveness in terms of identifying LS cases, the DGS protocol provided the best sensitivity (99.90%, 95% CI: 99.57–99.93%) and similar specificity (99.50%, 97.28–99.85%) compared to IHC (sensitivity 80.56%, 73.81–81.97% and specificity 99.98%, 99.89–99.99%) and MSI (sensitivity 82.50%, 76.15–84.03% and specificity 99.99%, 99.92–100.00%) protocols. TSGS protocol also provided better sensitivity (99.42%, 96.55–99.63%) but a slight less specificity (96.58%, 96.46–96.60%) compared to IHC and MSI protocols. MSI to germline sequencing improves sensitivity (85.04%, 79.40–86.82%) compared to the MSI protocol. Adding double somatic analysis to IHC, MSI, and MSI to germline sequencing does not change the sensitivity and specificity of the protocols in terms of identifying LS cases.

The protocol sensitivities and specificities translated to observations that in a hypothetical cohort of 1000 newly diagnosed CRC population with best estimate of 30 LS cases [18], DGS and TSGS protocols would identify all the LS cases with 0% cases missed. MSI to germline sequencing would identify 26 of the 30 LS patients, missing 13% of the cases. In comparison, IHC and MSI protocols would identify 24 and 25 LS patients respectively, missing 17–20% of the cases (Table 1). According to base-case best estimate, adding double somatic testing reduced the number of unexplained cases by 70% from 44 to 13 for IHC, and by 50% from 27 to 13 for MSI and 102 to 51 for MSI to germline sequencing (Table 1).

The total protocol costs for the hypothetical cohort of 1000 were $0.94M (95% CI: $0.51M–$1.69M) for DGS protocol, compared to $0.49M ($0.38M–$0.60M) for IHC, $0.57M ($0.44M–$0.71M) for MSI, $0.61M ($0.47M–$0.79M) for MSI to germline sequencing, and $3.11M ($2.26M–$3.94M) for TSGS protocol. Adding double somatic testing, total protocol costs increased to $0.52M ($0.40M–$0.64M) for IHC, $0.59M ($0.46M–$0.73M) for MSI, and $0.68M ($0.55M–$0.86M) for MSI to germline sequencing (Table 1).

Based on cost per LS case identified, the IHC protocol was the most efficient protocol at $20,082 compared to DGS protocol ($31,365), MSI protocol ($22,988), MSI to germline sequencing protocol ($23,726) and TSGS protocol ($104,394) (Table 1).

One-way sensitivity analysis showed that for DGS protocol, the cost of germline genetic sequencing had the greatest impact on the efficiency (Supplementary Materials Figure S2). For TSGS protocol, the cost of tumor sequencing had the greatest impact on the efficiency (Supplementary Materials Figure S2). Additional one-way sensitivity analyses for other protocols are included in Supplementary Materials Figure S2.

### 3.2. Threshold Analysis

Threshold analysis demonstrated that the cost of the germline sequencing panel to the healthcare system would need to be $368 for DGS protocol to be as efficient as the IHC protocol. And the cost of tumor sequencing test would need to drop to $508 for the TSGS protocol to reach the same efficiency as the IHC protocol.

## 4. Discussion

This study developed multiple decision analytical models representing eight current and potential near-future LS screening protocols for identifying LS cases among newly diagnosed CRC populations. The models were developed to support further discussion about which protocol is most appropriate for implementation in healthcare systems for LS case identification based on new evidence. We believe one of the key issues for many healthcare systems is whether the time has arrived to consider a DGS protocol or a TSGS protocol which are simpler and have superior LS case-finding potential, and may represent more realistic real-world clinical workflows. Instead of reporting traditional incremental cost-effectiveness ratios in economic evaluation modeling which can be difficult to interpret by decision-makers [11], our model enabled examination of detailed outcome metrics including effectiveness, cost, and efficiency that were deemed important and easy to interpret for healthcare system decision-making based on our prior studies [11,29].

Our findings suggest that the DGS and TSGS protocols were most effective, i.e., identified the most LS cases and missed the fewest LS cases, followed by the MSI to germline sequencing protocol, compared to traditional IHC and MSI protocols. Our reported sensitivities and specificities of the modeled LS screening protocols in terms of identifying LS cases were consistent or within a reasonable range when compared with the literature [18]. We found the sensitivities and specificities of the IHC and MSI protocols were lower and higher, respectively, in our analysis than what is reported in Hampel et.al. (2018) where IHC plus *BRAF* had sensitivity of 89.7% (78.8–96.1%) and MSI plus *BRAF* had sensitivity of 91.4% (81.0–97.1%), and IHC plus *BRAF* had specificity of 94.6% (91.9–96.6%), and MSI plus *BRAF* had specificity of 94.8% (92.2–96.8%) [18]. These variances can be explained by differences in the IHC and MSI protocols applied-in our models we included both *BRAF* and promoter hypermethylation testing, whereas Hampel et.al. (2018) included IHC/MSI plus only *BRAF* in the sensitivity and specificity calculations. In addition, since the sensitivity and specificity for tumor sequencing were directly adopted from Hampel et al. (2018), we also note that there may be differences in the test performance of germline sequencing in conjunction with the tumor sequencing.

Even with the reduction in the market price of germline genetic sequencing in recent years, our findings were still consistent with prior literature conclusions that the IHC protocol was the most economically efficient [9,13,30]. In our case, the IHC protocol was the most efficient ($20,082 per LS case identified), though can miss up to 17–20% more LS cases compared to other modeled protocols. Prior study showed IHC plus *BRAF* and methylation protocol costs $10,693 per LS case identified [11]. The difference was generated from the fact that the costs used in prior work were based on an internal reference laboratory of Intermountain Healthcare (as the study was specifically to inform local decision-making at that healthcare system) rather than the Medicare fee schedule, and the reported costs are in 2010 US dollars versus in 2018 US dollars in this study.

Threshold analysis showed that DGS protocol would be as efficient as the IHC protocol if the cost of germline sequencing declines to $368 or less. The current market price of DGS ranges widely from around $250 to typically over $2000 billed to healthcare system or insurance. To some extent, DGS might already be an optimal option to certain healthcare systems depending on the negotiated price. Given the continuing declines in costs of most germline genetic tests, DGS could be an efficient LS screening approach in the near future. With over 6 times the cost per LS case identified compared to the IHC protocol, the emerging combined approach of tumor and germline sequencing is not an efficient protocol solely for the purpose of LS case identification. The addition of double somatic testing to IHC and MSI protocols slightly increased protocol costs (e.g., by 6% or $32 per CRC case screened for IHC protocol), but reduced the number of unexplained cases by 70% (IHC protocol) and 50% (MSI and MSI to germline sequencing protocols). This could decrease inappropriate costs and risks for unnecessary surveillance for patients and inappropriate costs for healthcare systems, but these scenarios and associated costs were not modeled. Before DGS cost declines to the estimated threshold, IHC and MSI protocols with double somatic testing would be the optimal choices for universal LS screening for the primary purpose of identifying individuals with LS.

This study has several limitations. First, common to typical decision analytical modeling studies, the model input and assumptions were based on general literature, thus the usefulness of the outcomes comparing the LS screening protocols in terms of supporting decision-making at a local healthcare system may be limited [31]. To help support local decision-making on which LS screening protocol would be most appropriate for implementation in a given healthcare system, model input and assumptions based on local data and circumstances are needed to evaluate site-specific outcomes. Our prior work demonstrates the feasibility of generic models to provide useful precision medicine economic evidence supporting local decision-making by allowing use of local-specific input values [32]. As a next step under the IMPULSS project, we converted the conventional decision analytical models developed in this study to a generic modeling tool to allow end-users to interact and enter parameter values and obtain model outputs specific to local healthcare systems [33]. Ultimately, together with information gathered and analyzed from other aims of IMPULSS, we will generate a “toolkit” for each participating healthcare system to use and guide local implementation, maintenance, and improvement of LS screening [26].

We recognize that in real-world practice, varying permutations of the IHC protocols may be implemented due to logistical and system abilities. In this study, we chose to model IHC including reflex testing to both *MLH1* promoter methylation and *BRAF* V600E testing as it is the approach best aligned with NCCN guidelines and existing evidence from literature [7,11,13]. It is beyond this study’s scope or intention to model all protocol permutations that could exist in the real-world of US healthcare systems.

Since this study is from a healthcare system perspective, one could argue that the cost of germline sequencing would be zero where healthcare systems do not pay for germline testing or get reimbursed for testing. This may be the case in many fee-for-service systems, however, in other models, such as integrated systems, this cost does exist for the healthcare system and may be an important factor in LS screening program implementation.

In addition, for simplicity in comparison of multiple LS screening approaches, we assumed 100% compliance with the LS screening protocols, which is unrealistic in real-world implementation. There are reports of loss to follow-up and thus failure in effectiveness of LS case identification in traditional multi-step IHC protocols. However, the reported compliance rate varies widely [20,21]. The consent rate for genetic sequencing in the DGS protocol is also not well studied in the US and the compliance rate could vary widely based on individual patient, clinician, and healthcare system factors.

The main goal of this study was to provide an updated general insight of the comparative outcomes of LS screening protocols; real-world implementation issues are beyond the scope of this study. However, our generic modeling tool as a next step will allow flexibility to consider and account for site-specific situations including varying permutations of the IHC protocols, costs, and compliance rates in real-world as mentioned above.

In the TSGS protocol, the test performance of the combination of tumor sequencing and germline sequencing was based on a published study [18] of a test that is not yet clinically available, and may not reflect the performance of the tests currently available in market due to lack of such information for these tests. Nonetheless, it was thought important to model this potential approach, given the likelihood that it could emerge into clinical practice. There are some caveats to the interpretation of the results of this model. The cost of tumor sequencing in this protocol may be overestimated as tumor sequencing is already utilized for all stage IV and many stage III CRC patients, thus there would be only incremental costs of adding MMR genes for these patients. However, while in theory the two sequencing tests (tumor and germline) may be ordered together for a one-time price, in clinical reality, additional tests may still be necessary, given the optimal tumor sequencing test proposed in the TSGS protocol with both MMR and prognostic biomarkers (KRAS, NRAS, and *BRAF*) is not yet clinically available. And information on incremental cost is also not available to model at this point; meaning that the model is heavily dependent on assumptions. However, there is extensive information available on the costs of testing that provides a reasonable extrapolated cost estimate for panels of 5–15 genes that would likely include a proposed panel to support TSGS testing.

Further limitation of the study is that we only focused on identification of probands with LS and does not include cascade testing among family members. Finally, our model only focused on comparing outcomes of multiple protocols in terms of LS case identification and did not model and compare the benefit of treatment guidance to targeted therapy. One could argue that even if DGS is adopted for LS case identification purpose, tumor analysis for evidence such as microsatellite instability may still be needed to guide treatment as a separate objective. To date, there is no consensus or standard of approach that addresses both objectives. Traditional multi-step IHC and MSI approaches are concerned with issues of missing LS cases and loss of follow up [18,19,20,21] and not particularly for treatment guidance. The TSGS protocol explored by Hampel et.al. [18] which did not “win out” in our model due to the high costs of the test, may yield better comparative outcomes when adding considerations of treatment guidance as it comprehensively addresses, in one or two steps, the dual objectives. However, upfront tumor sequencing still misses more LS cases compared to germline sequencing and requires sequential germline sequencing to confirm LS cases. In addition, expensive and comprehensive tumor sequencing is only relevant for patients with stage III and IV tumors for treatment guidance purposes, thus, its universal use is not expected to be an efficient approach. A better approach might be upfront germline sequencing followed by tumor examination. In sum, models comparing different protocols addressing both objectives of LS case identification and treatment guidance to provide evidence supporting more comprehensive clinical decision-making are warranted as an important and novel next step once clinical protocols begin to emerge into practice.

## 5. Conclusions

Based on this modeling study, while tumor screening with IHC remains the most efficient approach to identify patients with LS, the decreasing cost of sequencing coupled with increased sensitivity is approaching a point where transitioning to a DGS approach should be seriously considered.

## Figures and Tables

**Table 1:** Base-case and probabilistic sensitivity analysis on outcome measures.

**Outcomes**	**DGS**		**IHC**		**MSI**		**MSIGS**	
	**Base Case**	**(95% CI)**	**Base Case**	**(95% CI)**	**Base Case**	**(95% CI)**	**Base Case**	**(95% CI)**
**Effectiveness**								
**Sensitivity of protocol**	99.90%	(99.57–99.93%)	80.56%	(73.81–81.97%)	82.50%	(76.15–84.03%)	85.04%	(79.40–86.82%)
**Specificity of protocol**	99.50%	(97.28–99.85%)	99.98%	(99.89–99.99%)	99.99%	(99.92–100.00%)	99.95%	(99.68–99.98%)
Number of true LS cases expected to be identified *	30	(23–37)	24	(17–30)	25	(18–30)	26	(19–31)
**Number of missed LS cases**	0	(0–0)	6	(5–9)	5	(4–8)	4	(4–7)
**Number of unexplained dMMR cases**	NA	NA	44	(36–46)	27	(19–33)	102	(98–128)
**Costs**								
**Cost per protocol for a 1000hypothetical cohort ($ millions)**	$0.94	($0.51–$1.69)	$0.49	($0.38–$0.60)	$0.57	($0.44–$0.71)	$0.61	($0.47–$0.79)
**Cost per CRC case screened**	$940	($514–$1687)	$485	($375–$598)	$569	($437–$710)	$605	($471–$788)
**Efficiency**								
**Cost per true LS case identified**	$31,365	($16,618–$60,814)	$20,082	($14,604–$29,676)	$22,988	($17,006–$33,986)	$23,726	($17,336–$35,748)
**Outcomes**	**IHCDS**		**MSIDS**		**MSIGSDS**		**TSGS**	
	**Base Case**	**(95% CI)**	**Base Case**	**(95% CI)**	**Base Case**	**(95% CI)**	**Base Case**	**(95% CI)**
**Effectiveness**								
**Sensitivity of protocol**	80.56%	(73.96–81.93%)	82.50%	(76.11–84.12%)	85.04%	(79.30–86.95%)	99.42%	(96.55–99.63%)
**Specificity of protocol**	99.98%	(99.88–99.99%)	99.99%	(99.93–100.00%)	99.95%	(99.68–99.98%)	96.58%	(96.46–96.60%)
Number of true LS cases expected to be identified *	24	(17–29)	25	(18–30)	26	(19–31)	30	(22–37)
**Number of missed LS cases**	6	(5–9)	5	(4–8)	4	(3–7)	0	(0–1)
**Number of unexplained dMMR cases**	13	(11–17)	13	(8–15)	51	(39–59)	43	(31–57)
**Costs**								
**Cost per protocol for a 1000 hypothetical cohort ($ millions)**	$0.52	($0.40–$0.64)	$0.59	($0.46–$0.73)	$0.68	($0.55–$0.86)	$3.11	($2.26–$3.94)
**Cost per CRC case screened**	$517	($405–$636)	$588	($460–$728)	$679	($547–$857)	$3114	($2263–$3940)
**Efficiency**								
**Cost per true LS case identified**	$21,396	($16,244–$30,736)	$23,771	($17,751–$33,880)	$26,624	($19,803–$39,286)	$104,394	($76,520–$150,355)

Note: * The number of true LS cases expected to be identified is 30 (20–40) in the hypothetical cohort of 1000 CRC patients based on LS prevalence of 3% (2–4%). LS, Lynch syndrome; CRC, colorectal cancer; DGS, direct germline sequencing; IHC, immunohistochemistry; MSI, microsatellite instability; MSIGS, MSI to germline sequencing; IHCDS, IHC with double somatic; MSIDS, MSI with double somatic; MSIGSDS, MSI to germline sequencing with double somatic; TSGS, tumor sequencing to germline sequencing.

## Data Availability

This study is an economic evaluation involves decision analytic modeling. All parameter values and assumptions used to support the model, which are mostly derived from published literature or expert opinion, are listed in the paper. The excel based decision analytic model is available on request.

## Supplementary Materials

The following are available online at https://www.mdpi.com/article/10.3390/jpm11121284/s1. Figure S1: Lynch Syndrome (LS) Screening Protocols Model Structure Diagrams; Figure S2: Multiple 1-Way Probabilistic Sensitivity Analysis on Efficiency of Modeled Lynch Syndrome Screening Protocol; Table S1: External Validation by Protocol.

## Appendix A

**Table A1:** Model Parameters.

Parameter	Base Case	Range Minimum	Range Maximum	Distribution	Protocols Affected	Reference
**Prevalence**						
Prevalence of Lynch Syndrome in CRC patients	3%	2%	4%	Beta	All	Palomaki 2009 [3]
**Compliance to Protocol**						
Genetic counseling and consenting	100%	100%	100%	-	All	Assumption
Collection of blood specimen for sequencing	100%	100%	100%	-	All	Assumption
Successful and reportable sequencing	100%	100%	100%	-	All	Assumption
Appropriate tumor tissue available and collected	100%	100%	100%	-	All IHC, All MSI, TSGS	Assumption
IHC test successful and reportable	100%	100%	100%	-	All IHC	Assumption
*BRAF* test successful and reportable	100%	100%	100%	-	All IHC, MSI, MSIDS	Assumption
Methylation test successful and reportable	100%	100%	100%	-	All IHC, MSI, MSIDS	Assumption
Double somatic test successful and reportable	100%	100%	100%	-	IHCDS, MSIDS, MSIGSDS	Assumption
MSI test successful and reportable	100%	100%	100%	-	All MSI	Assumption
**Probabilities**						
Probability of IHC positive result	14.70%	14.20%	14.70%	Beta	All IHC	Hampel 2008 [25], Expert opinion based on Hampel 2018 (supplementary table) [18]
Probability of *MLH1* absence result (of IHC positive result)	70.00%	67.61%	73.60%	Beta	All IHC	Hampel 2008 [25], Expert opinion based on Hampel 2018 (supplementary table) [18], Palomaki 2009 [3]
Probability of LS genetic test positive result (of IHC positive without *MLH1* absence)	60.00%	54.20%	68.30%	Beta	All IHC	Hampel 2008 [25], Expert opinion based on Hampel 2018 (supplementary table) [18]
Probability of double somatic test positive result (of IHC positive, *MLH1* absent, LS genetic testing negative)	95.00%	89.00%	96.00%	Beta	IHCDS	Pearlman 2019 [34], Haraldsdottir 2014 [22]
Probability of double somatic test positive result (of IHC positive without *MLH1* absence, LS genetic testing negative)	57.00%	42.00%	64.00%	Beta	IHCDS	Pearlman 2019 [34], Haraldsdottir 2014 [22]
Probability of MSI high result	12.80%	12.58%	18.14%	Pert	All MSI	Hampel 2008 [25], Expert opinion based on Hampel 2018 (supplementary table)
Probability of double somatic test positive result (of MSI-high, LS genetic testing negative)	50.00%	50.00%	80.00%	Pert	MSIDS, MSIGSDS	Pearlman 2019 [34], Geurts-Giele 2014 [35], Haraldsdottir 2014 [22]
Probability of double somatic positive result at tumor sequencing	3.30%	3.30%	3.30%	Pert	TSGS	Hampel 2018 [18]
**Test Performance**						
Sensitivity of Next Generation Sequencing (NGS) panel	99.90%	99.50%	100.00%	Beta	All	Expert panel opinion, * Pritchard 2012 [36], Gallego 2015 [37]
Specificity of Next Generation Sequencing (NGS) panel	99.50%	95.00%	100.00%	Pert	All	Expert panel opinion, * Pritchard 2012 [36]
Sensitivity of IHC test	83.00%	75.00%	89.00%	Beta	All IHC	Palomaki 2009 [3]
Specificity of IHC test	88.80%	67.60%	94.80%	Beta	All IHC	Palomaki 2009 [3]
Sensitivity of MSI test	85%	77%	89%	Beta	All MSI	Palomaki 2009 [3]
Specificity of MSI test	90.20%	85%	94%	Beta	All MSI	Palomaki 2009 [3], Mvundura 2010 [13]
Sensitivity of tumor sequencing test	99.50%	93.80%	100%	Pert	TSGS	Hampel 2018 [18]
Specificity of tumor sequencing test	95.30%	92.60%	97.20%	Pert	TSGS	Hampel 2018 [18]
PPV *BRAF* test (following IHC)	99.00%	99.00%	99.00%	-	All IHC	Expert panel opinion *
NPV *BRAF* test (following IHC)	21.67%	21.67%	21.67%	-	All IHC	Expert opinion based on Hampel 2018 (supplementary table) [18]
PPV Methylation test (following IHC)	99.00%	99.00%	99.00%	-	All IHC	Expert panel opinion *
NPV Methylation test (following IHC)	38.24%	38.24%	38.24%	-	All IHC	Expert opinion based on Hampel 2018 (supplementary table) [18]
PPV *BRAF* test (following MSI)	99.00%	99.00%	99.00%	-	MSI, MSIDS	Expert panel opinion *
NPV *BRAF* test (following MSI)	36.36%	36.36%	36.36%	-	MSI, MSIDS	Expert opinion based on Hampel 2018 (supplementary table) [18]
PPV Methylation test (following MSI)	99.00%	99.00%	99.00%	-	MSI, MSIDS	Expert panel opinion *
NPV Methylation test (following MSI)	48.00%	48.00%	48.00%	-	MSI, MSIDS	Expert opinion based on Hampel 2018 (supplementary table) [18]
**Costs**						
Cost of genetic counseling time per patient	$220.00	$110.00	$330.00	Gamma	All	Medicare Fee Schedule 2018 [38], Expert panel opinion *
Cost of genetic sequencing panel	$720.00	$250.00	$2600.00	Gamma	All	Medicare Fee Schedule 2018 [39], Expert panel opinion, * market patient price amounts [27,28]
Cost of IHC screen	$395.00	$197.50	$592.50	Gamma	All IHC	Medicare Fee Schedule 2018 [38], Expert panel opinion *
Cost of *BRAF* test	$175.00	$87.50	$262.50	Gamma	All IHC, MSI, MSIDS	Medicare Fee Schedule 2018 [39], Expert panel opinion *
Cost of methylation of *MLH1* promoter test	$190.00	$95.00	$285.00	Gamma	All IHC, MSI, MSIDS	Medicare Fee Schedule 2018 [39], Expert panel opinion *
Cost of double somatic test	$725.00	$362.50	$1087.50	Gamma	IHCDS, MSIDS, MSIGSDS	Medicare Fee Schedule 2018 [38,39], Expert panel opinion *
Cost of MSI test	$485.00	$242.50	$727.50	Gamma	All MSI	Medicare Fee Schedule 2018 [38,39], Expert panel opinion *
Cost of tumor sequencing test	$3045.00	$1522.26	$4566.78	Gamma	TSGS	Medicare Fee Schedule 2018 [38,39], Expert panel opinion *

* Based on the IMPULSS clinical expert panel which is teamed with clinical experts of the IMPULSS study team from eight participating healthcare systems and the IMPULSS External Advisory Board. LS, Lynch syndrome; CRC, colorectal cancer; DGS, direct germline sequencing; IHC, immunohistochemistry; MSI, microsatellite instability; MSIGS, MSI to germline sequencing; IHCDS, IHC with double somatic; MSIDS, MSI with double somatic; MSIGSDS, MSI to germline sequencing with double somatic; TSGS, tumor sequencing to germline sequencing.

## Appendix B

### Calculation of Sensitivity and Specificity to Support Modeling

All test sensitivity and specificity values in the models were based on the detection of LS cases. Since sensitivity and specificity values for *BRAF* and *MLH1* promoter hypermethylation tests reported in the literature refer to test performance in identifying somatic changes [3] and not in terms of detection of LS cases, we back-calculated the sensitivity and specificity values based on their positive predictive value (PPV) and negative predicted value (NPV) and prevalence of LS at relevant points in the models. For modeling purposes, we defined PPV as true negative LS/*BRAF* (methylation) test positive, and NPV as true positive LS/*BRAF* (methylation) test negative. The PPV and NPV values in these terms were based on expert opinion from Ms. Heather Hampel based on data analysis from the Hampel 2018 (supplementary table) for *BRAF* and methylation testing following IHC (*MLH1* absence) and following MSI (high) [18]. The values and ranges of the PPV and NPV were further informed by clinical expert opinion based on evidence in the literature that LS is highly unlikely to be detected in those with positive *BRAF* V600E variant testing and methylation testing in the *MLH1* region among CRC cases with *MLH1* protein absence or MSI (high) [3,8,17,18,40,41,42].

In probabilistic sensitivity analysis, we applied correlation coefficients of −0.8 and −0.9 for sensitivity and specificity pairs generated for IHC and MSI tests respectively, based on receiver operating characteristic curves found in de Freitas et al. [43]. We did not include correlation between sensitivity and specificity for germline sequencing tests due to insufficient data and in line with expert opinion to apply a wider range with lower specificity values and maintain a higher, more narrow range for sensitivity.

## References

[1] Collins F.S., Varmus H. (2015). A New Initiative on Precision Medicine. N. Engl. J. Med..

[2] Healthy People 2020: Genomics. https://www.healthypeople.gov/2020/topics-objectives/topic/genomics/objectives.

[3] Palomaki G.E., McClain M.R., Melillo S., Hampel H.L., Thibodeau S.N. (2009). EGAPP supplementary evidence review: DNA testing strategies aimed at reducing morbidity and mortality from Lynch syndrome. Genet. Med..

[4] Siegel R.L., Miller K.D., Goding Sauer A., Fedewa S.A., Butterly L.F., Anderson J.C., Cercek A., Smith R.A., Jemal A. (2020). Colorectal cancer statistics, 2020. CA Cancer J. Clin..

[5] National Cancer Institute Cancer Moonshot. Blue Ribbon Panel Report 2016. Sept 7, 2016. https://www.cancer.gov/research/key-initiatives/moonshot-cancer-initiative/blue-ribbon-panel/blue-ribbonpanelreport-2016.pdf.

[6] CDC Public Health Genomics: Genomic Tests and Family Health History by Levels of Evidence. https://phgkb.cdc.gov/PHGKB/topicFinder.action?Mysubmit=init&query=tier+1.

[7] National Comprehensive Cancer Network (2016). NCCN Guidelines: Genetic/familial high-risk assessment: Colorectal (version 1.2016). J. Natl. Compr. Cancer Netw..

[8] Berg A.O., Armstrong K., Botkin J., Calonge N., Haddow J., Hayes M., Kaye C., Phillips K.A., Piper M., Richards C.S. (2009). Recommendations from the EGAPP Working Group: Genetic testing strategies in newly diagnosed individuals with colorectal cancer aimed at reducing morbidity and mortality from Lynch syndrome in relatives. Genet. Med..

[9] Di Marco M., DAndrea E., Panic N., Baccolini V., Migliara G., Marzuillo C., De Vito C., Pastorino R., Boccia S., Villari P. (2018). Which Lynch syndrome screening programs could be implemented in the “real world”? A systematic review of economic evaluations. Genet. Med..

[10] Goverde A., Spaander M.C., van Doorn H.C., Dubbink H.J., Ouweland A.M.V.D., Tops C.M., Kooi S.G., de Waard J., Hoedemaeker R.F., Bruno M.J. (2016). Cost-effectiveness of routine screening for Lynch syndrome in endometrial cancer patients up to 70 years of age. Gynecol. Oncol..

[11] Gudgeon J.M., Williams J.L., Burt R.W., Samowitz W.S., Snow G., Williams M.S. (2011). Lynch syndrome screening implementation: Business analysis by a healthcare system. Am. J. Manag. Care.

[12] Leenen C.H.M., Goverde A., de Bekker-Grob E.W., Wagner A., van Lier M.G.F., Spaander M.C.W., Bruno M.J., Tops C.M., Ouweland A.M.W.V.D., Dubbink H.J. (2016). Cost-effectiveness of routine screening for Lynch syndrome in colorectal cancer patients up to 70 years of age. Genet. Med..

[13] Mvundura M., Grosse S.D., Hampel H., Palomaki G.E. (2009). The cost-effectiveness of genetic testing strategies for Lynch syndrome among newly diagnosed patients with colorectal cancer. Genet. Med..

[14] Le D.T., Uram J.N., Wang H., Bartlett B.R., Kemberling H., Eyring A.D., Skora A.D., Luber B.S., Azad N.S., Laheru D. (2015). PD-1 Blockade in Tumors with Mismatch-Repair Deficiency. N. Engl. J. Med..

[15] Giardiello F.M., Allen J.I., Axilbund J.E., Boland C.R., Burke C.A., Burt R.W., Church J.M., Dominitz J.A., Johnson D.A., Kaltenbach T. (2014). Guidelines on genetic evaluation and management of Lynch syndrome: A consensus statement by the US Multi-society Task Force on colorectal cancer. Am. J. Gastroenterol..

[16] Hampel H., de la Chapelle A. (2013). How do we approach the goal of identifying everybody with Lynch Syndrome?. Fam. Cancer.

[17] Hampel H., Frankel W.L., Martin E., Arnold M., Khanduja K., Kuebler P., Nakagawa H., Sotamaa K., Prior T.W., Westman J. (2005). Screening for the Lynch Syndrome (Hereditary Nonpolyposis Colorectal Cancer). N. Engl. J. Med..

[18] Hampel H., Pearlman R., Beightol M., Zhao W., Jones D., Frankel W.L., Goodfellow P.J., Yilmaz A., Miller K., Bacher J. (2018). Assessment of Tumor Sequencing as a Replacement for Lynch Syndrome Screening and Current Molecular Tests for Patients With Colorectal Cancer. JAMA Oncol..

[19] Bellcross C.A., Bedrosian S.R., Daniels E., Duquette D., Hampel H., Jasperson K., Joseph D.A., Kaye C., Lubin I., Meyer L.J. (2012). Implementing screening for Lynch syndrome among patients with newly diagnosed colorectal cancer: Summary of a public health/clinical collaborative meeting. Genet. Med..

[20] Gudgeon J.M., Varner M.W., Hashibe M., Williams M.S. (2019). Is immunohistochemistry-based screening for Lynch syndrome in endometrial cancer effective? The consent’s the thing. Gynecol. Oncol..

[21] Cragun D., DeBate R.D., Vadaparampil S.T., Baldwin J., Hampel H., Pal T. (2014). Comparing universal Lynch syndrome tumor-screening programs to evaluate associations between implementation strategies and patient follow-through. Genet. Med..

[22] Haraldsdottir S., Hampel H., Tomsic J., Frankel W.L., Pearlman R., de la Chapelle A., Pritchard C.C. (2014). Colon and Endometrial Cancers with Mismatch Repair Deficiency Can Arise From Somatic, Rather Than Germline, Mutations. Gastroenterology.

[23] Mensenkamp A.R., Vogelaar I.P., van Zelst-Stams W.A., Goossens M., Ouchene H., Hendriks-Cornelissen S.J., Kwint M.P., Hoogerbrugge N., Nagtegaal I.D., Ligtenberg M.J.L. (2014). Somatic mu-tations in MLH1 and MSH2 are a frequent cause of mismatch-repair deficiency in Lynch syndrome-like tumors. Gastroen-Terology.

[24] Sourrouille I., Coulet F., Lefevre J.H., Colas C., Eyries M., Svrcek M., Bardier-Dupas A., Parc Y., Soubrier F. (2012). Somatic mosaicism and double somatic hits can lead to MSI colorectal tumors. Fam. Cancer.

[25] Hampel H., Frankel W.L., Martin E., Arnold M., Khanduja K., Kuebler P., Clendenning M., Sotamaa K., Prior T., Westman J.A. (2008). Feasibility of Screening for Lynch Syndrome Among Patients With Colorectal Cancer. J. Clin. Oncol..

[26] Rahm A.K., Cragun D., Hunter J.E., Epstein M.M., Lowery J., Lu C.Y., Pawloski P., Sharaf R.N., Liang S.-Y., Burnett-Hartman A.N. (2018). Implementing universal Lynch syndrome screening (IMPULSS): Protocol for a multi-site study to identify strategies to implement, adapt, and sustain genomic medicine programs in different organizational contexts. BMC Health Serv. Res..

[27] Color (Hereditary Cancer Panel). https://www.color.com/learn/can-cancer-be-inherited.

[28] Myriad (Colaris) Test Quotation via Phone Call to the Company on 30 October 2018.

[29] Gudgeon J.M., Belnap T.W., Williams J.L., Williams M.S. (2013). Impact of age cutoffs on a lynch syndrome screening program. J. Oncol Pract..

[30] Ladabaum U., Wang G., Terdiman J., Blanco A., Kuppermann M., Boland C.R., Ford J., Elkin E., Phillips K.A. (2011). Strategies to identify the Lynch syndrome among patients with colorectal cancer: A cost-effectiveness analysis. Ann. Intern. Med..

[31] Sculpher M.J., Pang F.S., Manca A., Drummond M.F., Golder S., Urdahl H., Davies L., Eastwood A. (2004). Generalisability in economic evaluation studies in healthcare: A review and case studies. Health Technol. Assess..

[32] Snyder S.R., Hao J., Cavallari L.H., Geng Z., Elsey A., Johnson J.A., Mohamed Z., Chaiyakunapruk N., Chong H.Y., Dahlui M. (2018). Generic Cost-Effectiveness Models: A Proof of Concept of a Tool for Informed Decision-Making for Public Health Precision Medicine. Public Health Genom..

[33] Hassen D., Hao J., Gudgeon J.M., Snyder S.R., Hampel H., Williams M.S., Lu C., Sharaf R.N., Schwiter R., Burnett-Hartman A. Building a user-friendly modeling tool to inform and guide decision-making for lynch syndrome screening at local healthcare systems. Proceedings of the 13th Annual Conference on the Science of Dissemination and Implementation.

[34] Pearlman R., Haraldsdottir S., De La Chapelle A., Jonasson J.G., Liyanarachchi S., Frankel W.L., Rafnar T., Stefansson K., Pritchard C.C., Hampel H. (2019). Clinical characteristics of patients with colorectal cancer with double somatic mismatch repair mutations compared with Lynch syndrome. J. Med. Genet..

[35] Geurts-Giele W.R.R., Leenen C.H.M., Dubbink H.J., Meijssen I.C., Post E., Sleddens H.F.B.M., Kuipers E.J., Goverde A., Ouweland A.M.W.V.D., Van Lier M.G.F. (2014). Somatic aberrations of mismatch repair genes as a cause of microsatellite-unstable cancers. J. Pathol..

[36] Pritchard C.C., Smith C., Salipante S.J., Lee M.K., Thornton A.M., Nord A., Gulden C., Kupfer S.S., Swisher E.M., Bennett R.L. (2012). ColoSeq Provides Comprehensive Lynch and Polyposis Syndrome Mutational Analysis Using Massively Parallel Sequencing. J. Mol. Diagn..

[37] Gallego C.J., Shirts B.H., Bennette C.S., Guzauskas G., Amendola L.M., Horike-Pyne M., Hisama F.M., Pritchard C.C., Grady W.M., Burke W. (2015). Next-Generation Sequencing Panels for the Diagnosis of Colorectal Cancer and Polyposis Syndromes: A Cost-Effectiveness Analysis. J. Clin. Oncol..

[38] Centers for Medicare & Medicaid Services (2018). Physician Fee Schedule 2018.

[39] Centers for Medicare & Medicaid Services (2018). Clinical Laboratory Fee Schedule 2018.

[40] Deng G., Peng E., Gum J., Terdiman J., Sleisenger M., Kim Y.S. (2002). Methylation of hMLH1 promoter correlates with the gene silencing with a region-specific manner in colorectal cancer. Br. J. Cancer.

[41] Kane M.F., Loda M., Gaida G.M., Lipman J., Mishra R., Goldman H., Jessup J.M., Kolodner R. (1997). Methylation of the hMLH1 promoter correlates with lack of expression of hMLH1 in sporadic colon tumors and mismatch repair-defective human tumor cell lines. Cancer Res..

[42] Kuismanen S.A., Holmberg M.T., Salovaara R., de la Chapelle A., Peltomäki P. (2000). Genetic and Epigenetic Modification of MLH1 Accounts for a Major Share of Microsatellite-Unstable Colorectal Cancers. Am. J. Pathol..

[43] de Freitas I.N., de Campos F.G., Alves V.A., Cavalcante J.M., Carraro D., Rde A.C., Diniz M.A., Nahas S.C., Ribeiro U. (2015). Proficiency of DNA repair genes and microsatellite instability in operated colorectal cancer patients with clinical suspicion of lynch syndrome. J. Gastrointest. Oncol..

