# Supplementary material for: Economic Evaluation of Universal Lynch Syndrome Screening Protocols among Newly Diagnosed Patients with Colorectal Cancer
Source: J Pers Med. 2021 Dec 2;11(12):1284. doi: 10.3390/jpm11121284 (PMC8708954; doi:10.3390/jpm11121284)
Supplement: Supplementary file 1 [file jpm-11-01284-s001.zip › jpm-1459767-supplementary.pdf]

Supplementary Materials

Figure S1. Lynch Syndrome (LS) Screening Protocols Model Structure Diagrams

A. Direct Germline Sequencing (DGS)

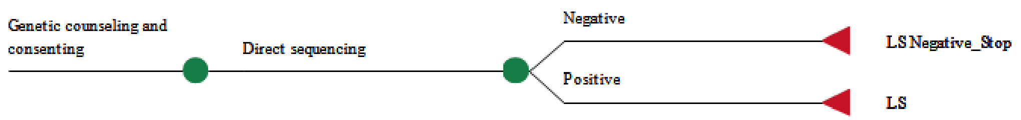

B. Immunohistochemistry (IHC)

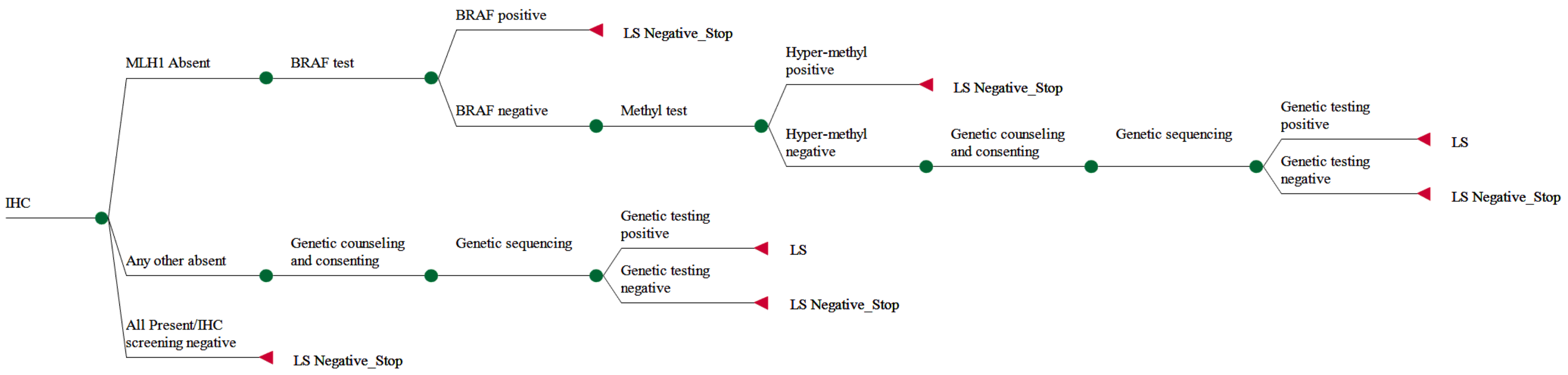

C. Microsatellite Instability (MSI)

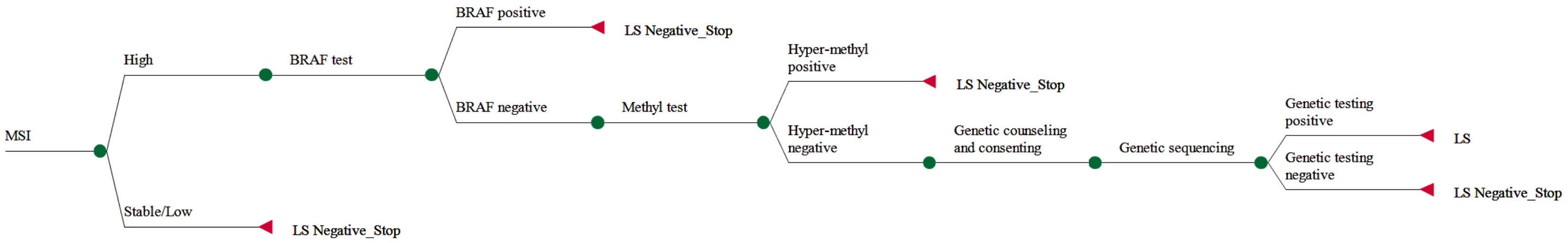

D. Microsatellite Instability to Germline Sequencing (MSIGS)

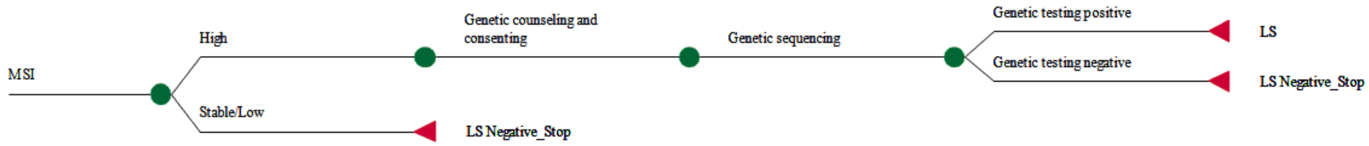

E. Immunohistochemistry with Double Somatic (IHCDS)

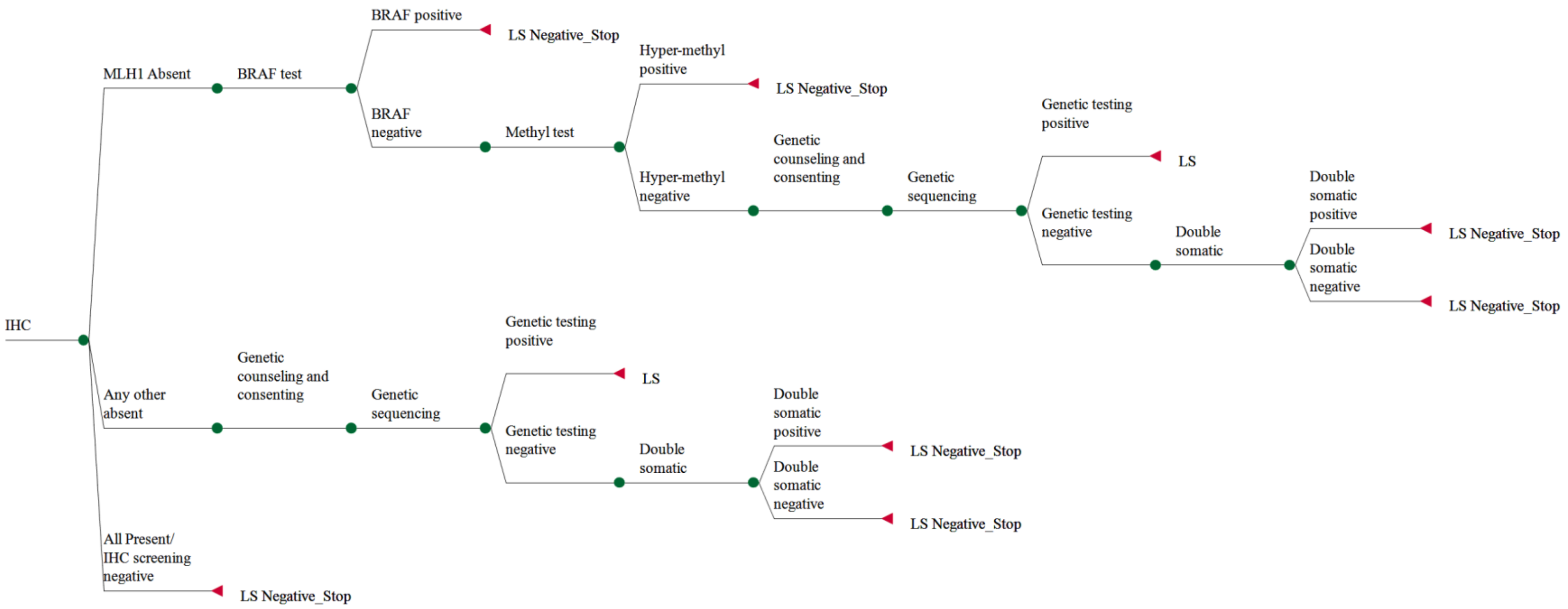

F. Microsatellite Instability with Double Somatic (MSIDS)

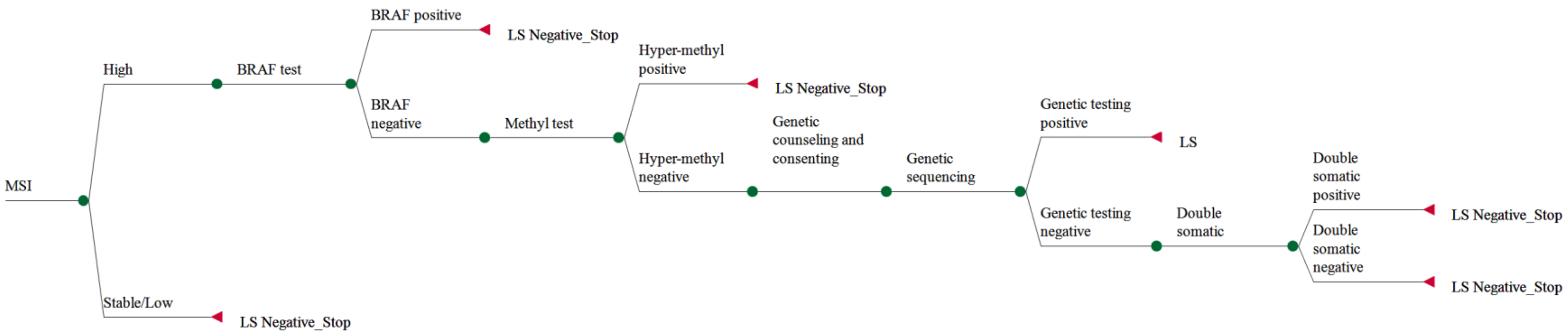

G. Microsatellite Instability to Germline Sequencing with Double Somatic (MSIGSDS)

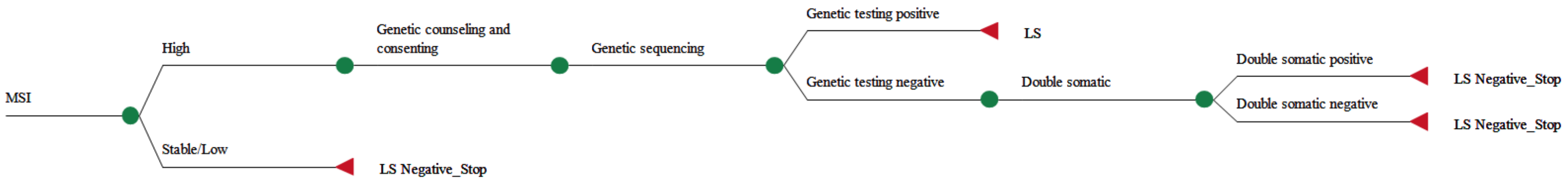

H. Tumor Sequencing to Germline Sequencing (TSGS)

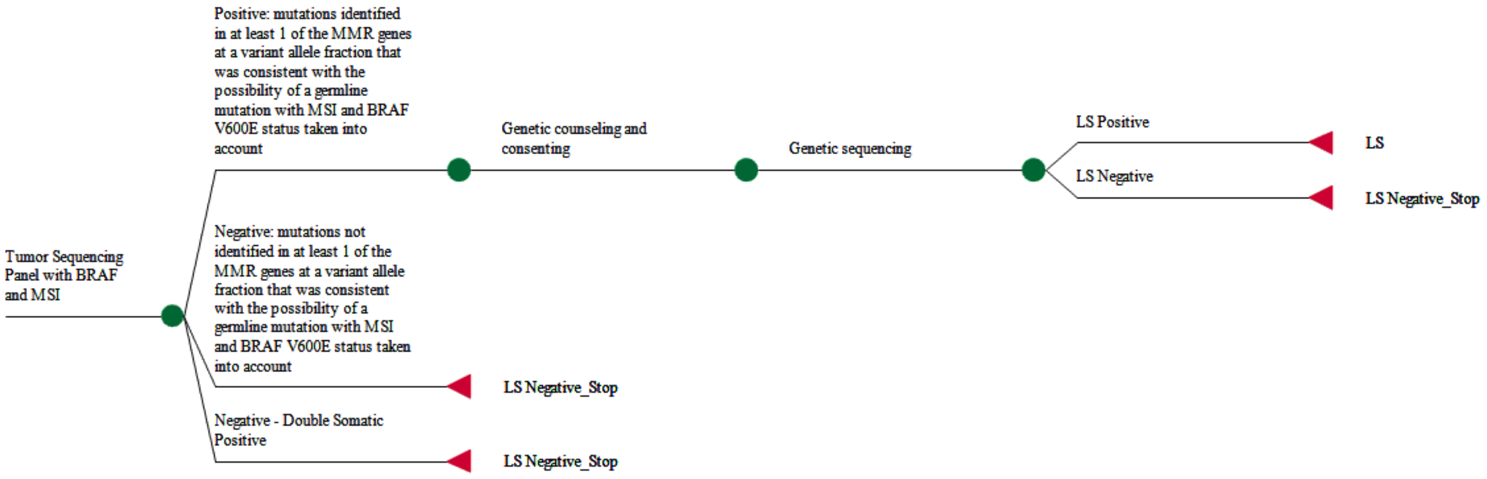

Table S1. External Validation by Protocol

External Validation Point by Protocol	Model Output Value	Reported Value(s) from the Literature			
**IHC**		**Value (1)**	**Reference (1)**	**Value (2)**	**Reference (2)**
Sensitivity of IHC Protocol (IHC plus BRAF)	80.56%	89.70% (78.8%-96.1%)	Hampel 2018[1]		
Specificity of IHC Protocol (IHC plus BRAF)	99.98%	94.60% (91.9%-96.6%)	Hampel 2018[1]		
Probability of *BRAF* positive result (out of IHC (MLH1 absent))	57.85%	60.00%	Hampel (unpublished data)[2]	68.33%	Hampel 2018 (supplementary table)[1]
Probability of PHM positive result (out of IHC (MLH1 absent), *BRAF* (neg))	44.50%	31.00%	Palomaki 2009[3]	42.11%	Hampel 2018 (supplementary table)[1]
Probability of LS genetic test positive result (out of IHC (MLH1 absent), *BRAF* (neg), PHM (neg))	38.51%	30.77%	Hampel 2008[4]	63.64%	Hampel 2018 (supplementary table)[1]
Probability of Double Somatic test positive result (out of IHC positive, LS genetic testing negative)	69.85%	57.00%-78.00%	Pearlman 2019[5]	70.97%	Haraldsdottir 2014[6]
**MSI**					
Sensitivity of MSI Protocol (MSI plus *BRAF*)	81.13%	91.4% (81.0%-97.1%)	Hampel 2018[1]		
Specificity of MSI Protocol (MSI plus *BRAF*)	99.99%	94.8% (92.2%-96.8%)	Hampel 2018[1]		
Probability *BRAF* positive result (out of MSI (high))	46.40%	56.58%	Hampel 2018 (supplementary table)[1]		
Probability PHM positive result (out of MSI (high), *BRAF* (neg))	24.77%	24.24%	Hampel 2018 (supplementary table)[1]		
Probability of LS positive genetic test result (out of MSI (high), *BRAF* (neg), PHM (neg))	48.21%	48.00%	Hampel 2018 (supplementary table)[1]		
**MSI to Germline Sequencing**					
Probability of LS positive genetic test result (out of MSI (high))	20.33%	15.79%	Hampel 2018[1]	28.13%	Hampel 2008[4]

Figure S2. Multiple 1-Way Probabilistic Sensitivity Analysis on Efficiency of Modeled Lynch Syndrome Screening Protocols

2A. Direct Germline Sequencing (DGS) Protocol

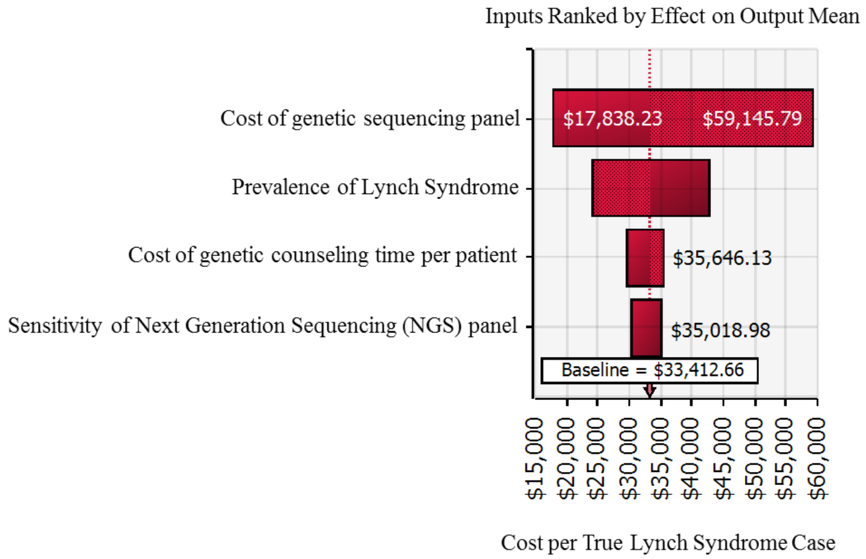

2B. Immunohistochemistry (IHC) Protocol

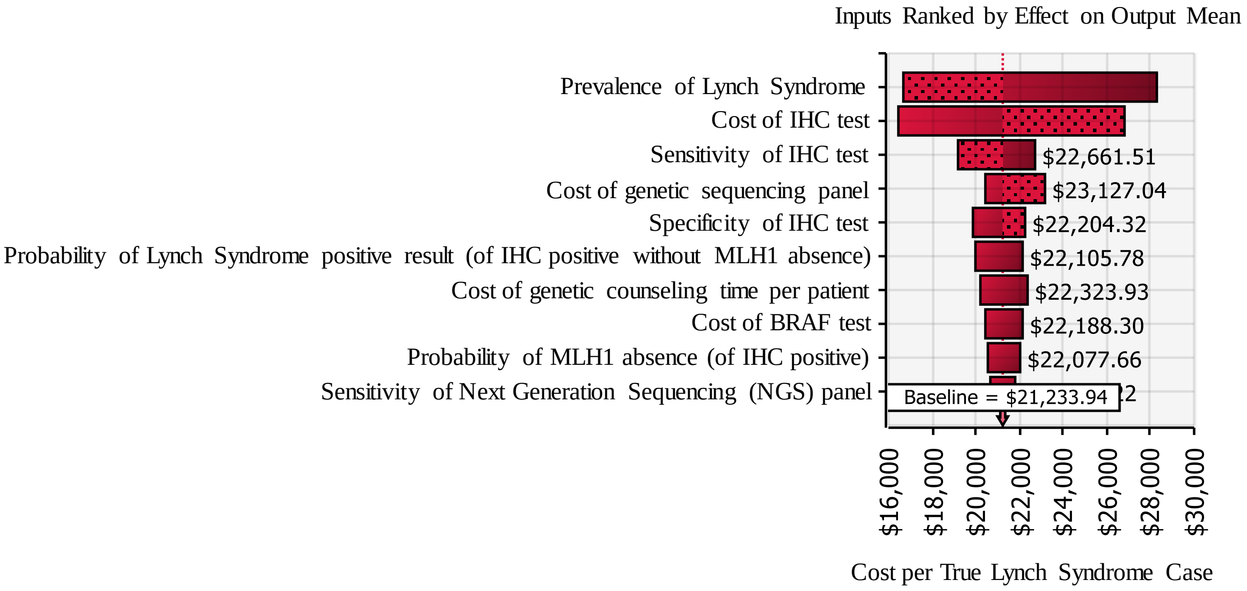

2C. Microsatellite Instability (MSI) Protocol

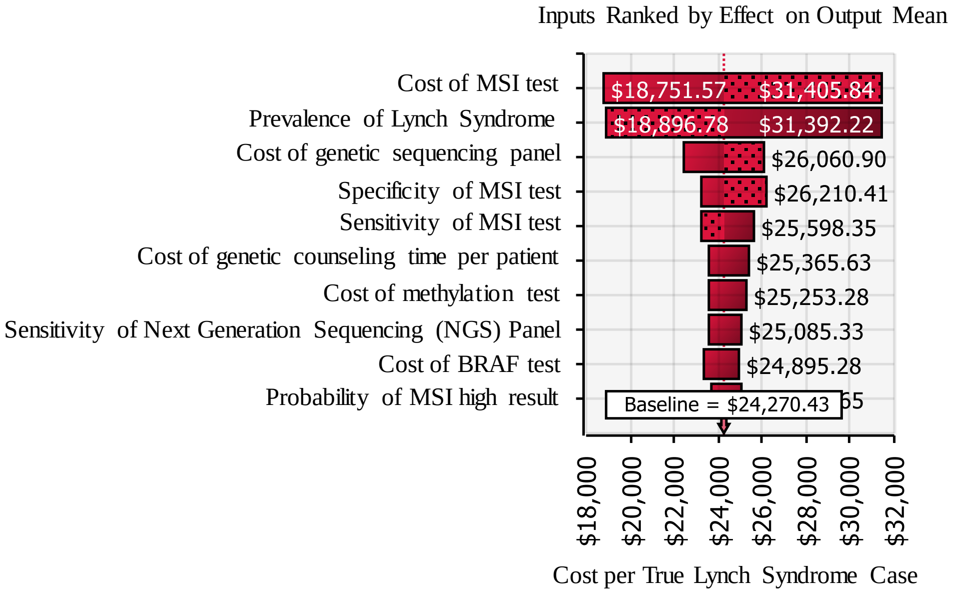

2D. Microsatellite Instability (MSI) to Germline Sequencing Protocol

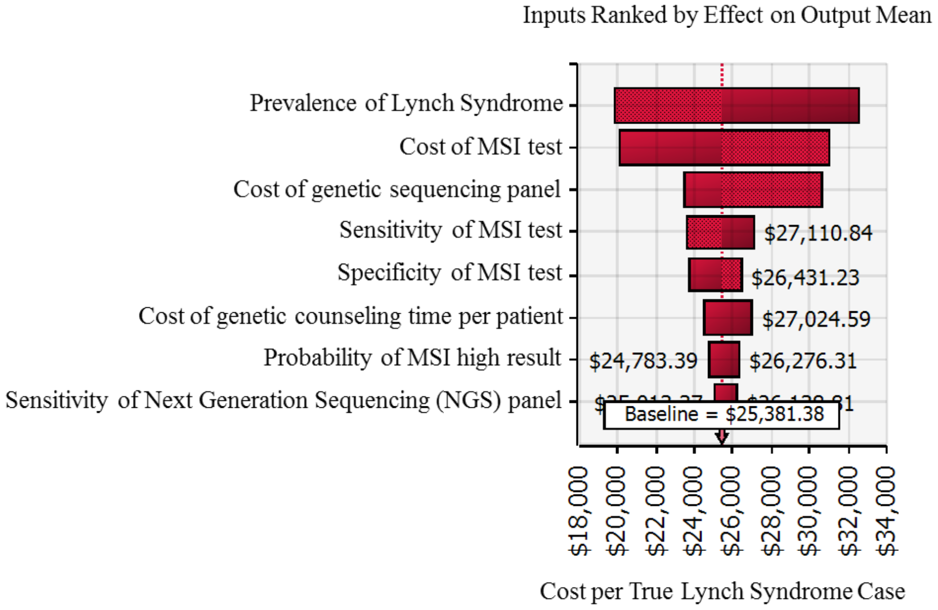

2E. Immunohistochemistry (IHC) with Double Somatic Protocol

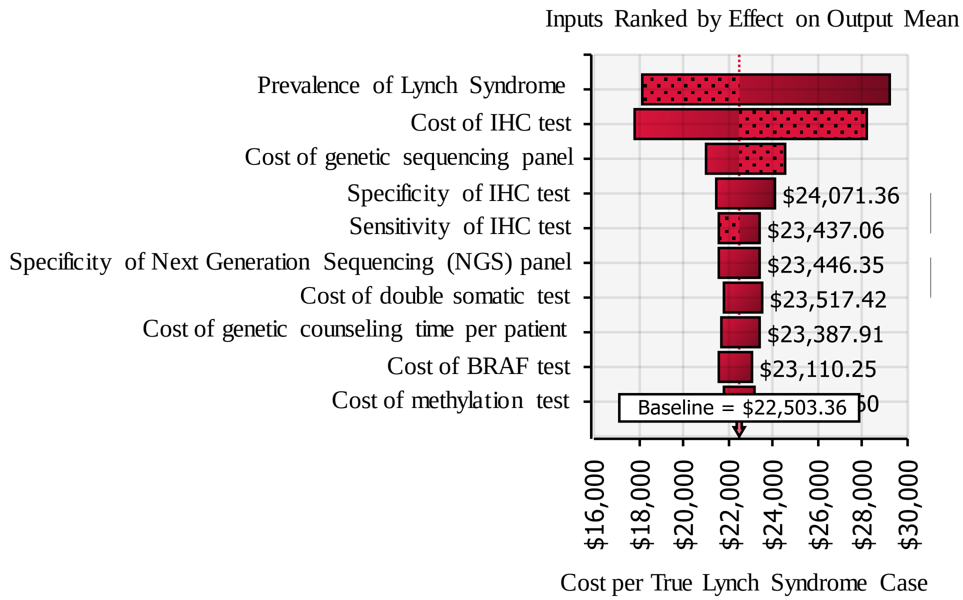

2F. Microsatellite Instability (MSI) with Double Somatic Protocol

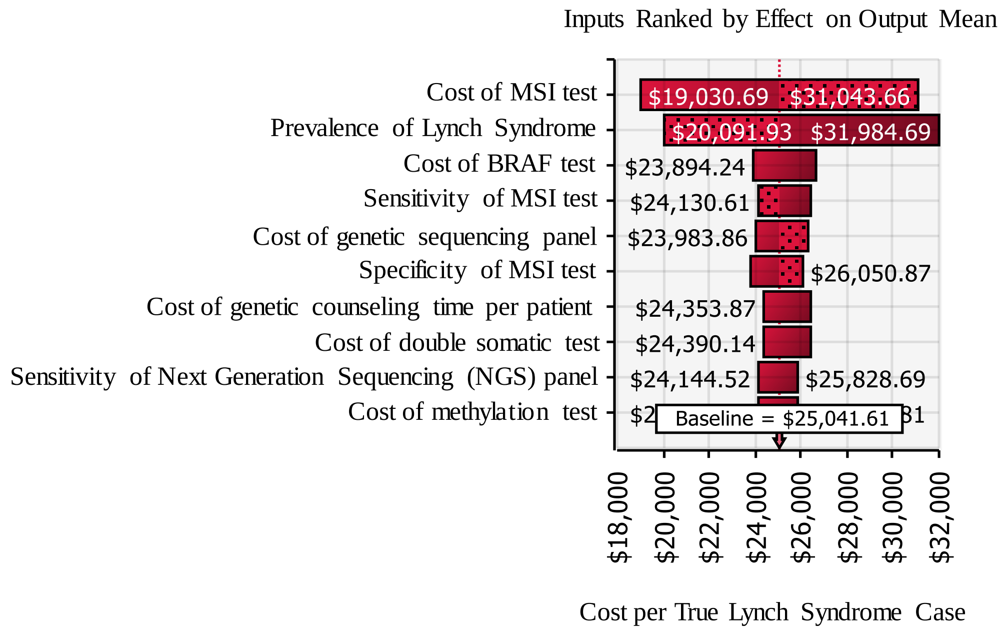

2G. Microsatellite Instability (MSI) to Germline Sequencing with Double Somatic Protocol

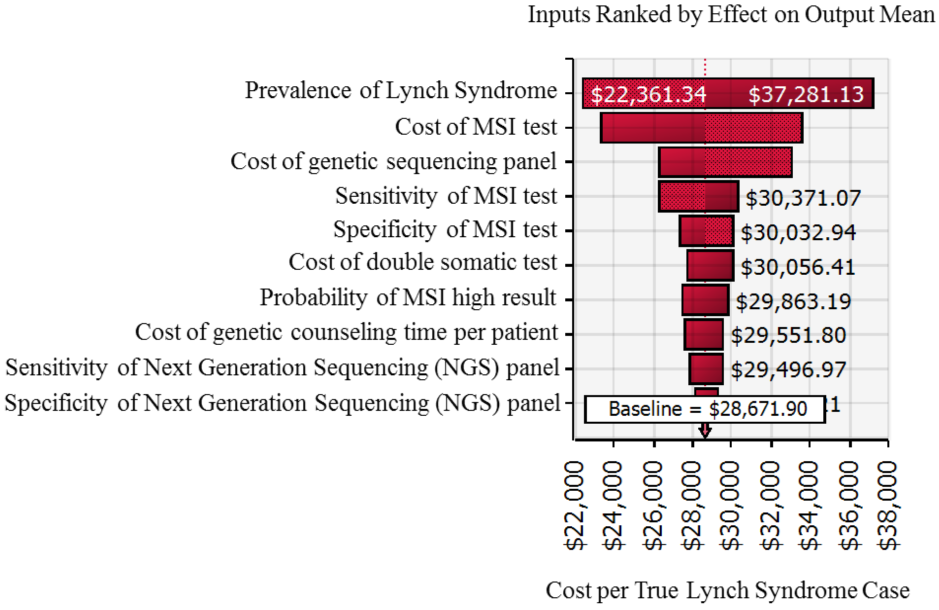

2H. Tumor Sequencing to Germline Sequencing (TSGS) Protocol

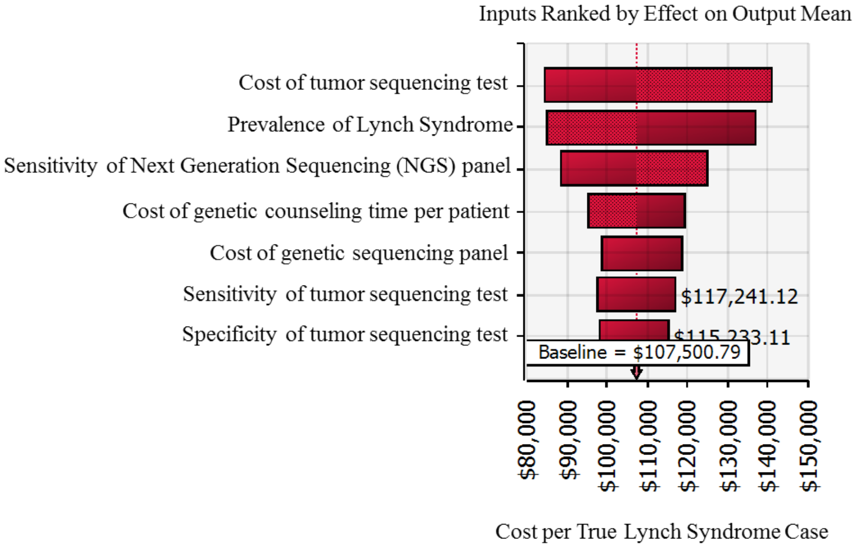

References

1. Hampel H, Pearlman R, Beightol M, Zhao W, Jones D, Frankel WL, et al. Assessment of Tumor Sequencing as a Replacement for Lynch Syndrome Screening and Current Molecular Tests for Patients With Colorectal Cancer. JAMA Oncol. 2018;4(6):806-13.
2. Hampel H. 2013-2016 Unpublished data.
3. Palomaki GE, McClain MR, Melillo S, Hampel HL, Thibodeau SN. EGAPP supplementary evidence review: DNA testing strategies aimed at reducing morbidity and mortality from Lynch syndrome. Genet Med. 2009;11(1):42-65.
4. Hampel H, Frankel WL, Martin E, Arnold M, Khanduja K, Kuebler P, et al. Feasibility of screening for Lynch syndrome among patients with colorectal cancer. J Clin Oncol. 2008;26(35):5783-8.
5. Pearlman R, Haraldsdottir S, de la Chapelle A, Jonasson JG, Liyanarachchi S, Frankel WL, et al. Clinical characteristics of patients with colorectal cancer with double somatic mismatch repair mutations compared with Lynch syndrome. J Med Genet. 2019;56(7):462-70.
6. Haraldsdottir S, Hampel H, Tomsic J, Frankel WL, Pearlman R, de la Chapelle A, et al. Colon and endometrial cancers with mismatch repair deficiency can arise from somatic, rather than germline, mutations. Gastroenterology. 2014;147(6):1308-16 e1.
